# The impact of COVID-19 pandemic on the incidence, presentation, and management of type 1 diabetes in children and adolescents: a narrative review

**DOI:** 10.1007/s42000-025-00662-2

**Published:** 2025-04-18

**Authors:** Irene Papapetrou, Agnieszka Swiecicka

**Affiliations:** 1 University of Nicosia Medical School, Nicosia, Cyprus; 2 Consultant in Endocrinology and Diabetes, Zoi Medical Centre, Nicosia, Cyprus

**Keywords:** Type 1 diabetes, COVID-19 pandemic, Children and adolescents, Incidence of type 1 diabetes, Diabetes management, Diabetic ketoacidosis

## Abstract

Type 1 diabetes (T1D) is an autoimmune condition affecting approximately 1.5 million children and adolescents worldwide, with an incidence of approximately 2–3% each year and rising. During the recent COVID-19 pandemic, a significant increase in incidence of T1D in children and adolescents was observed in numerous countries worldwide, with an increased number of newly-diagnosed cases presenting with diabetic ketoacidosis. The increased frequency of T1D presenting with diabetic ketoacidosis has been attributed not only to the SARS-CoV-2 virus itself but also to the restrictions imposed by the pandemic. The shift to telemedicine and unwillingness to seek medical care due to fear of infection contributed to delayed diagnosis and more severe disease presentation. Furthermore, the periods of lockdown that were implemented during the pandemic presented multiple challenges for children and adolescents living with T1D and disrupted the management of their condition. Changes in physical activity and diet as well as shortage of medical supplies during that period have been linked to worsening of glycemic control, which were at least partly offset by increased parental involvement and use of telemedicine.

## Introduction

Type 1 diabetes (T1D) is a chronic autoimmune condition characterized by destruction of pancreatic beta-cells resulting in deficiency of insulin and subsequent hyperglycemia [1]. Approximately 1.5 million children and adolescents younger than 20 years old currently live with T1D globally [2] and the incidence of the condition appears to be increasing by 2–3% each year, with the largest increase observed in children less than 15 years old [3].

Significant variations in incidence of T1D have been observed across various ethnic groups and geographical regions, with the highest incidence rates seen in the Caucasian populations of Northern European origin, namely, Finland (52.2 per 100,000 per year), Sweden (44.1 per 100,000 per year), and Norway (33.6 per 100,000 per year). Asian and African nations have significantly (24 to 50 times) lower incidence rates of T1D, although studies are to date lacking for the majority of countries [4].

A number of factors have been implicated in the development of T1D, with genetic predisposition considered to play a critical role. While the general population has a 0.4% lifetime risk of developing T1D, this increases to 7% when an individual has a sibling diagnosed with T1D [5]. Genomic studies have identified more than 60 genes associated with T1D, with the Human Leukocyte Antigen (HLA) accounting for up to 50% of genetic risk [6].

In addition, environmental factors such as diet, diversity of intestinal microbiota, and exposure to viruses in early life [3], specifically coxsackie virus B, cytomegalovirus, Epstein-Barr, and varicella zoster virus, have been more closely linked with new onset T1D [7].

In contrast, exclusive breastfeeding for more than 4 months [6], maternal intestinal and vaginal microbiome [8], exposure to probiotics in early infancy, and vitamin D sufficiency, among others, have been found to be protective against the development of islet cell autoimmunity [9].

Diabetic ketoacidosis (DKA) represents one of the most common acute complications of T1D [10] and remains the leading cause of morbidity and mortality in this patient population [11]. Ketoacidosis develops when ketone bodies (byproducts of lipid metabolism) build up in the blood as a result of uninhibited lipolysis, which is caused by extremely low levels of insulin [12]. DKA can be the initial presenting feature of T1D or it may arise in individuals already diagnosed with diabetes [13].

An international retrospective study, conducted between 2006 and 2016, on DKA at T1D diagnosis in 59,000 children reported an overall average DKA prevalence of 29.9%; it also noted a significant geographic variation, with increased prevalence in countries closer to the equator and in those with a lower human development index [13]. Similar geographic variation in the frequency of DKA presenting at T1D diagnosis across countries was confirmed in a systematic review of 29,000 children in 31 countries across five continents, reporting DKA frequencies ranging from 12.8 to 80% [14]. Interestingly, countries with the highest incidence of T1D globally, such as the Scandinavian countries [4, 15] and Canada [4], report lower frequencies of DKA at T1D diagnosis compared to countries with typically lower incidence rates of T1D, such as Romania, Italy, Luxembourg, Slovenia, the USA, and Taiwan [4, 13, 14], reflecting a varying degree of clinical suspicion.

New-onset T1D presenting with DKA has been associated with poor long-term glycemic control [16–18] and an increased mortality rate of 4.3% [19] compared to an overall mortality rate of < 0.35% in developed countries [20]. Early detection and treatment of DKA remains critical in young patients due to an increased risk of serious complications, such as cerebral edema, rhabdomyolysis, thrombosis, and stroke [21]. Cerebral edema, which might affect up to 5% of young diabetic patients with DKA, is associated with a mortality rate of 20–50% [11].

Insulin therapy stands as the cornerstone of medical treatment for T1D and can be administered through basal-bolus regimens utilizing long-acting and rapid-acting insulin analogues, or continuous subcutaneous insulin infusion (CSII) therapy. Children diagnosed with T1D require specialized care, thorough diabetes education, anticipatory guidance, and counseling on healthy behavior so that their developmental and emotional needs, as well as their family’s, are met [22]. Furthermore, a number of additional unique aspects of medical care exist in this population and pose additional challenges to healthcare providers, such as patient supervision in childcare settings or schools, changes in insulin sensitivity related to physical growth and sexual maturation, and neurological vulnerability to hypoglycemia and hyperglycemia in young children [23].

Coronavirus disease (COVID-19) is a respiratory illness caused by a virus known as severe acute respiratory syndrome coronavirus 2 (SARS-CoV-2). COVID-19 typically presents as an upper respiratory infection, the symptoms being cough, fever, and fatigue [24]. Less commonly, the disease manifests with gastrointestinal symptoms such as vomiting and diarrhea [25]. Pulmonary symptoms of COVID-19 can progress to pneumonia and/or acute respiratory distress syndrome due to the affinity of the virus to the angiotensin-converting enzyme-2 (ACE2) receptors located in pulmonary alveolar cell type II.

These receptors, beyond being expressed in the lungs, kidneys, pancreas, and gastrointestinal tract [26], are also found in cardiac cells, which could explain the cardiac manifestations associated with the illness, such as myocarditis, cardiomyopathies, and heart failure [27].

The illness displays varying degrees of severity, as highlighted in a notable case-series study conducted by the Chinese Center for Disease Control and Prevention [28]. According to their findings, 81% of those infected had a mild form of the illness, while 14% experienced more severe symptoms such as dyspnea, respiratory rate above 30 per min, and blood oxygen saturation below 93%. Furthermore, 4.7% developed critical illness, characterized by respiratory failure, septic shock, and/or multiple organ failure [28]. Patients with comorbidities were at increased risk of severe illness [27], those with COVID-19 and concurrent diabetes representing almost 40% of the critically ill cohort [29].

In March 2020, the World Health Organization (WHO) declared COVID-19 a pandemic [30]. The COVID-19 pandemic spread round the globe within weeks, disrupting many aspects of life, such as health, education, availability of food and jobs, and transportation [31]. To date, the virus has infected more than 600 million people and has caused more than 6 million deaths worldwide [30].

In an attempt to control transmission of the virus, governments around the world have implemented a large number of restrictions, such as quarantines, social distancing, closure of schools, universities, companies, and factories [31], banning flights from affected areas, closure of borders, early testing for COVID-19 and tracking of contacts with the aim of limiting social interaction [24]. Furthermore, in-person visits to physicians were limited and occurred only in situations where it was deemed absolutely necessary, with many physicians adopting the use of telemedicine [32].

During the first year of the pandemic, a significant 9.5% increase in the incidence of pediatric new-onset T1D was observed worldwide [33], with delays in seeking medical attention for children presenting with signs and symptoms of T1D linked to this rise in incidence [34] as well as to an increase in newly diagnosed T1D presenting with DKA [35]. Additionally, restrictions imposed during the pandemic disrupted routine healthcare for young patients managing T1D and their caregivers [36].

This review aims to assess the published literature investigating the effect of the COVID-19 pandemic on the incidence, presentation, and management of T1D in children and adolescents.

## Methods

For the purpose of this narrative review, a search for studies published in the English language was conducted through PubMed using different combinations of the keywords “type 1 diabetes,” “new-onset,” “children,” “adolescents,” “COVID-19 pandemic,” “incidence,” “management,” and “diabetic ketoacidosis.” The Boolean operators “AND” and “OR” were used in between each keyword to ensure generation of relevant published literature. All types of studies published between 2010 and 2024 were considered.

## T1D in children and adolescents

T1D represents the most common form of diabetes in the pediatric population [23], with a peak incidence in children aged 10–14 years, although it can manifest at any age [1]. Patients will typically present with polyuria, polydipsia, unintentional weight loss, and fatigue; however, the first presentation with DKA related to absolute or relative insulinopenia is not an uncommon clinical scenario [12].

The presentation of T1D with DKA has been attributed to delayed diagnosis or misdiagnosis, predominantly due to parental or physician error in recognizing classic symptoms of the condition [37]. It has been reported that 55% of children with new-onset T1D presenting with DKA were originally misdiagnosed with other more common conditions, such as viral/bacterial infections or gastroenteritis, thereby delaying treatment [11].

A systematic review of 46 studies involving more than 24,000 children from 31 countries with new onset T1D reported that age below 10 years, ethnic minority status, lower body mass index, and history of preceding infection were all associated with an increased risk of presenting with DKA at T1D diagnosis [38].

The increased risk of DKA in younger children has been attributed to underdeveloped mechanisms of metabolic compensation in response to dehydration and acidosis [38–40], while language and cultural barriers as well as lack of T1D awareness could also possibly explain the observed increased risk in ethnic minorities [13, 38].

A Korean multicenter study involving 361 children with new onset T1D linked a history of preceding infection and lower parental education to an increased risk of severe (venous blood pH < 7.1) DKA at the time of diagnosis [41]. It has been postulated that infection might trigger accelerated autoimmune destruction of the pancreatic beta-cells, while the symptoms of the infection might mask the early symptoms of T1D leading to a diagnostic delay [38, 41].

T1D is treated using an individualized insulin regimen that takes into consideration several factors, such as the child’s age, lifestyle, support at school, and personal preferences [22]. Components that can impact adherence to treatment and require frequent assessment include education of daycare providers and school nurses, behavior and emotional state of the young patient, and psychosocial factors such as peer relationships, school performance, and depression or anxiety [23]. Unlike adults, children progress through several developmental phases in their life; it is therefore important for appropriate diabetic care and education to be routinely performed throughout these transitions [22].

In addition to medical treatment, nutrition and physical activity are vital components for the management of T1D. Young patients with T1D need an individualized nutrition therapy, taking into consideration their food preferences, schedules, family habits, and physical activity while concurrently educating the patients and their families on how to monitor their carbohydrate intake in order to maintain good glycemic control [23].

The demanding multidisciplinary management of diabetes can become a significant burden for young patients and their families, with many children and adolescents displaying signs of psychosocial maladjustment after their diagnosis [42]. When compared with healthy children and adolescents, those with T1D are more likely to develop depression, stress-related mental disorders, anxiety, and eating disorders, all of which have been linked to poor glycemic control [10]. Psychological interventions in these cases have been found to be very effective in improving not only mental health and quality of life but also overall diabetes control [22].

## Incidence of new-onset T1D during COVID-19

Data on T1D incidence, prevalence, and mortality in children and adolescents is limited and varies significantly among countries [2]. The WHO DIAMOND project examined worldwide incidence of T1D in children younger than 14 years old between 1990 and 1999 and reported an overall age-adjusted incidence ranging from 0.1 cases per 100,000 per year in China and Venezuela to 40.9 cases per 100,000 per year in Finland [43]. This variability has been attributed to differences in case ascertainment and lack of data from countries with a low gross domestic product and high mortality rates among children under the age of 5, who succumb to infectious diseases before T1D symptoms manifest. Furthermore, during the study period, an increase in incidence in T1D cases of 2.8% was observed [43].

Given the lack of data on the burden of T1D, Gregory et al. devised modeled estimates for incidence and prevalence of the condition in 2021. They estimated 510,000 new T1D diagnoses worldwide, of which 194,000 were children and adolescents less than 19 years old. In total, 8.42 million people worldwide were estimated to have T1D and of those 1.48 million were younger than 19 years [2]. Similarly, the International Diabetes Federation Atlas examined population-based studies on T1D incidence in the pediatric population and estimated that 149,500 children and adolescents under 20 years were diagnosed with T1D in 2021 worldwide. However, more than half the countries covered in this study did not have their own incidence data, requiring extrapolation from nearby countries [4].

During the COVID-19 pandemic, many countries worldwide reported a significant increase in incidence of T1D in the pediatric population [33, 44–49]. For example, a multicenter regional UK study in children up to the age of 16 years reported an 80% increase in the incidence of T1D between March and June, 2020, compared to the same time period before the pandemic [44]. Similarly, an Italian study reported a 77% increase in new onset T1D within the pediatric population up to 14 years old between October 2020 and April, 2021, compared to the same pre-pandemic period [45].

In Romania, the National Diabetes Register showed an average annual increase of 0.8% in incidence of pediatric T1D between 2015 and 2019; however, during the first year of the pandemic this incidence increased by 16.9% [46]. Similarly, a cross-sectional retrospective US study of children with new onset T1D showed a 57% increase in incidence of T1D during the first year of the pandemic, between March 2020 and 2021 compared to annual rates in the preceding 5 years [47].

An Egyptian cross-sectional study involving children and adolescents less than 18 years old showed a 27.4% increase in the number of newly diagnosed T1D during the first and second COVID-19 wave compared to the same pre-pandemic time period [48]. Furthermore, a multicenter retrospective study of 3,062 Polish children aged 0–18 years with new onset T1D reported an increase in T1D incidence from 21.55 cases per 100,000 person-years during the pre-pandemic period to 25.90 cases per 100,000 person-years during the first year of the pandemic [49].

In contrast, a retrospective Jordanian single-center study of 137 children younger than 18 years with new onset T1D reported a significant decrease in incidence of T1D during the first year of the pandemic (March 2020-March 2021) compared to the previous year [50]. Similarly, an Italian cross-sectional study of 53 pediatric diabetes centers reported a 23% reduction in new-onset T1D in children younger than 15 years during the early phase of the COVID-19 pandemic, between February and April 2020 compared to the same period in 2019 [51]. The decreased incidence was attributed not only to a fear of SARS-CoV-2 exposure but also to a decreased exposure to seasonal viruses, a known precipitating factor for T1D [51].

A similar retrospective multicenter cohort study in Saudi Arabia of 260 children younger than 14 years with T1D reported similar frequency of new onset T1D between the first year of the pandemic and the year preceding it [52].

Lastly, the Canadian study by Ho et al. investigating new onset T1D in children younger than 18 during the first year of the pandemic concluded that the incidence of T1D was not significantly different during the pandemic when compared with the previous year [53].

A systematic review and meta-analysis of 26 studies involving children with new onset T1D worldwide reported a global incidence rate of T1D diagnosis of 19.73 per 100,000 children younger than 18 years in the year before the pandemic and an increased incidence rate of 32.39 per 100,000 in the first year of the pandemic [33].

The US cohort study evaluating the risk of new-onset T1D after SARS-CoV-2 infection reported that individuals below the age of 18 years who were infected with the virus were more likely than the controls to develop T1D 30 days post-infection (HR = 2.66) [54]. By contrast, two studies from Egypt and Italy reported that only a minority of patients with new onset T1D either tested positive or had a history of recent SARS-CoV-2 infection [48, 55].

The observed overall increase in incidence of T1D during the COVID-19 pandemic has been largely attributed to the SARS-CoV-2 virus itself.

The putative mechanisms include the potential precipitation of T1D in patients with pre-existing islet cell autoimmunity and direct beta-cell damage from the SARS-CoV-2 virus [56]. The virus has also been implicated in induction of pancreatic autoimmunity, which could potentially result in T1D development years after the initial infection [33, 56, 57].

Individuals with pre-existing pancreatic autoimmunity may experience an accelerated onset of T1D following COVID-19 due to enhanced, indirect beta-cell destruction from cytokine release and activation of CD8 + T-cells. Additionally, SARS-CoV-2 has been found to directly infect pancreatic beta-cells leading to acute beta-cell damage and insulin deficiency [56]. Similarly, acute pancreatitis was also observed in 1.8% of pediatric patients infected with COVID-19 during the pandemic [58].

It has been proposed that induction of pancreatic autoimmunity by SARS-CoV-2 may occur via bystander activation [56, 57] whereby the immune system responds to the viral infection by triggering a “cytokine storm” which causes localized inflammation. As a result, dendritic cells produce self-antigens mimicking SARS-CoV-2 antigens, which in turn activate autoreactive T-cells, leading to pancreatic damage consistent with pancreatitis [57]. However, this autoimmune process is slow and its long-term impact on T1D prevalence remains uncertain [56]. Future prospective cohort studies are required to assess whether COVID-19 infection increases the risk of pancreatic autoimmunity over time.

Angiotensin-converting enzyme 2 (ACE2) is a protein involved in the renin-angiotensin system (RAS) by converting the vasoconstrictor angiotensin II to angiotensin 1–7, which promotes vasodilation and inhibits vasoconstriction. Therefore, it plays a critical role in blood pressure regulation and cardiovascular function [59].

Expression of ACE2 was identified in various human organs and tissues, including the pancreas [60, 61]. Muller et al. demonstrated ACE2 expression in both exocrine and endocrine cells of the pancreas, this forming an entry point for SARS-CoV-2. The infection was associated with reduced numbers of insulin-secretory granules in beta-cells and impaired glucose-stimulated insulin secretion [26].

Lastly, damage to pancreatic vasculature by COVID-19 is another proposed mechanism through which the virus may lead to new onset T1D [57]. SARS-CoV-2 infection has been shown to cause microthrombi formation and fibrosis in adult human pancreatic cells [62].

The data on the incidence of new onset T1D during the pandemic and its association with SARS-CoV-2 are heterogeneous. Further large prospective studies are needed to help elucidate these inconsistencies.

## Presentation of T1D during COVID-19

A number of studies reported an increased incidence of new onset T1D presenting with ketoacidosis during the pandemic [32, 49, 52, 53, 55, 63–67]. An Italian study of 4,237 children < 18 years old with newly diagnosed T1D observed an increase of 3.7% in frequency of DKA and an increase of 3.8% in frequency of severe DKA (pH < 7.1 or bicarbonate < 5mmol/L) at the time of diagnosis during the COVID-19 pandemic in 2020 when compared to 2017–2019 [55].

Similarly, a Polish 2-year cross-sectional multicenter study of 3,062 patients < 18 years old with new-onset T1D reported an increase of 11.9% in incidence of DKA at disease presentation during the COVID-19 period between 2020 and 2021 when compared to the same period, pre-pandemic, in 2019–2020. Severe DKA was noted in 21.1% of cases [49].

In Spain, a multicenter study of 1,444 children aged < 14 years old reported a significant (12%) increase in cases of new-onset T1D presenting with DKA during the pandemic period (2020–2021), compared to the pre-pandemic period (2015–2019). Furthermore, 36% of those cases were classified as severe DKA [66].

Another US multicenter study of newly diagnosed T1D patients aged 0–26 years old identified an increase of 4.3% in the proportion of cases presenting in DKA during the pandemic when compared with the previous year. Of those, 15.4% were considered to represent severe DKA [63].

In Turkey, a retrospective study of 997 pediatric patients less than 18 years old identified an 8.6% increase in admissions with new-onset T1D presenting with DKA and a significant increase of 13.7% in patients presenting with severe DKA during the pandemic. Moreover, the study reported that approximately 70% of these patients tested positive for SARS-CoV-2 [68].

Similarly, the National UK Survey, undertaken by the UK Association of Children’s Diabetes Clinicians, reported 51% of children and adolescents diagnosed with new-onset T1D during the first months of the pandemic (*N* = 450) presented in DKA, in contrast to 38% before the pandemic [64]. Of these, 28% were found to be in severe DKA.

In addition to an increased frequency of DKA at T1D diagnosis during the pandemic, an increase in severity of DKA was evident, with some studies identifying those younger than 6 years of age to be at particularly high risk [32, 52, 53, 69, 70]. Furthermore, Salmi and colleagues reported retrospectively an increase in the incidence rate of pediatric intensive care unit admissions with severe DKA from 2.89 per 100,000 person-years in 2016–2019 to 9.38 per 100,000 person-years in 2020 [71].

Finally, a meta-analysis of 20 observational studies, including almost 65,000 pediatric patients with T1D assessed before and during the pandemic, showed a 44% increase in the risk of DKA in newly diagnosed T1D during the pandemic compared to the pre-pandemic period [67].

Increased incidence of DKA has been in part attributed to delayed presentation of new onset T1D stemming from unwillingness to seek medical attention due to fear of infection [48, 52, 64, 66, 67], limited healthcare services, and misdiagnosis [64]. A survey of healthcare professionals looking after pediatric patients with diabetes during COVID-19 pandemic from 215 diabetes centers worldwide reported delayed diagnosis of T1D in 22% of participating centers. This was attributed to caregivers’ avoidance of contact with the healthcare team due to fear of infection [72].

In the National UK Survey, delayed presentation was documented in 20% of cases and attributed to fear of contracting the virus as well as inability to access specialist care in a timely manner [64]. Delayed presentation has also been reported in a small sample in Egypt where children presenting with DKA arrived at the emergency department 3 to 4 days after the onset of symptoms [65].

Furthermore, in certain health systems, regular diabetes screening testing performed in schools was cancelled during the pandemic, which might have contributed to the delayed diagnosis and severe disease presentation [34].

Conversely, several studies [32, 55, 73, 74] have reported similar duration of symptoms in patients with new onset T1D who presented with DKA prior to and during the pandemic, concluding that the delay in T1D diagnosis may not alone account for the increased risk for DKA during the pandemic [67].

Interestingly, one study reported that having a first degree relative diagnosed with diabetes who was able to recognize the signs and symptoms of the condition and seek medical help promptly was associated with a decreased risk of DKA presentation during the pandemic [50]. These results were in contrast to those of the earlier Canadian study which did not show a significant difference in the rate of DKA at T1D presentation among children with first degree relatives with diabetes [53].

In summary, the data indicate that there was an increase in incidence of T1D presenting with DKA, including severe DKA, in the pediatric population during the pandemic. Among others, decreased use of healthcare services and limited in-person visits to healthcare providers, which was replaced by telemedicine, were identified as possible contributory factors [53].

In addition, COVID-19 infection itself has been linked to increased insulin resistance, primarily due to inflammatory responses that impair glucose metabolism, leading to higher insulin requirements and increased risk of DKA in newly diagnosed T1D patients [75].

## Management of T1D during COVID-19

Optimal management of T1D depends on regular glucose monitoring, regular insulin administration, a healthy diet, and physical activity [76]. The pandemic and consequent lockdown posed significant challenges in the lives of T1D patients that inevitably affected management of their condition [48].

Several studies reported worsening of glycemic control in children and adolescents during lockdown, evidenced by increased levels of HbA1c [77–79] and more frequent hyper- and hypoglycemic episodes [78]. Worsening dietary habits, reduction in physical activity, and increase in “screen time” [79, 80] as well as irregular sleep patterns [79] were considered the major culprits [80].

A cross-sectional study of 100 T1D patients < 18 years old with a disease duration of > 1 year identified a significant increase in mean HbA1c levels, rising from 8.55 ± 1.63% before lockdown to 8.87 ± 1.61% after lockdown, this reportedly being associated with reduced physical activity, irregular meal timing, and irregular sleep and waking patterns [77].

Similarly, an Egyptian questionnaire-based study of 115 children and adolescents < 18 years old reported a percentage increment in HbA1c of 6.85 ± 1.67% after lockdown in 64% of patients, worse dietary and eating habits in 60% of patients, and more frequent hyper- and hypo-glycemic attacks in 66% and 59% of patients, respectively [78].

Another cross-sectional study of 52 children and adolescents reported a significant increase of 13.64% in HbA1c post-lockdown when compared to pre-lockdown, partly attributed to missed insulin doses and limited blood glucose monitoring owing to unavailability of supplies [79].

The outbreak of the pandemic in 2019 created shortages of insulin and glucose monitoring supplies in several countries [76, 79, 81] mainly attributable to disrupted deliveries after closure of healthcare and transport facilities [76], restricted supply in semi-urban and rural locations [79], and inability to access healthcare facilities due to curfew and lockdown [81]. Verma et al. reported that 50% of patients who had hyperglycemia during lockdown did not receive their insulin injections due to non-availability [79]. Moreover, fear of possible shortages has also been linked to infrequent glucose monitoring in several studies [78, 82].

A questionnaire-based cross-sectional study of 235 children and adolescents with T1D aged under 21 reported that insulin shortages compelled 14% of families to ration insulin and 43.4% to ration glucose test strips. Consequently, 75.5% of children who rationed glucose test strips experienced more frequent hypo- and hyperglycemia, while 54.5% of those who rationed insulin experienced hyperglycemia. Interestingly, rationing did not affect HbA1c levels when the latter was measured after lockdown; however, the authors attributed this to a small sample size and the duration of glucose variability which may not have be long enough to affect HbA1c values [81].

In contrast, several studies have associated the lockdown with improved glycemic control [83–87]. An Italian study involving 233 pediatric T1D patients aged 2–18 years using continuous glucose monitoring (CGM) observed a 4.9% reduction in HbA1c and a 10.3% increase in time-in-range (TIR, 70 and 180 mg/dL) post-lockdown compared to pre-lockdown [83].

Similarly, a retrospective observational study of 139 children and adolescents with T1D reported improved glycemic control post-lockdown, particularly among those with previously poor glycemic control [87].

In a study of 66 T1D children and adolescents using CGM, TIR increased by 4.7% and time above range (TAR > 180 mg/dL) decreased by 6.9% after 90 days of lockdown despite a reduction in physical activity [84]. Being away from a stress-generating school environment coupled with more accurate adjustments of insulin doses under closer parental supervision might be the factors contributing to observed improvements in glycemic control.

Furthermore, another study in Italy of 22 pre-school and school children with T1D < 18 years old reported a significant improvement in TIR of 5% and decreased TAR of 16.3% during the first two weeks of lockdown [85].

In Spain, a retrospective observational study of 80 children and adolescents with T1D who used CGM or flash interstitial glucose monitoring (FMG) systems reported improved glycemic control during the last 2 weeks of lockdown, particularly in children who exhibited higher HbA1c levels (≥ 7%) before lockdown and in those who were treated with multiple daily insulin injections. Interestingly, patients managed with continuous subcutaneous insulin infusion (CSII) who exhibited good glycemic control before lockdown maintained good control during lockdown, with a significant decrease in the time spent in the hypoglycemic range (< 70 mg/dL), suggesting adequate adjustment of the automated basal insulin infusion to the demands of lockdown [86].

Enforced home confinement but with more regularity in management of insulin therapy were the factors considered to be associated with better long-term glycemic control.

Moreover, it has been demonstrated that the majority of patients were able to maintain a healthy and balanced diet, adhere to regular physical activity, and monitor their glucose carefully thanks to greater awareness of their condition and appropriate use of diabetes technology [80]. In addition, parents have reported that during the pandemic they were able to be more actively involved in management of their child’s condition in that they had better control of their diet, physical activity, and glucose levels [80]. This was particularly evident in the case of younger (< 12 years old) patient groups [88].

As for the effects of lockdown on glycemic control in adolescents, the results were heterogeneous. Increased exposure to parental figures has been shown to have had a negative impact on glycemic control in pubertal adolescents compared to younger pre-pubertal patients [89]. This has been attributed to the physical and emotional changes experienced in adolescence as well as the transition to independence, making this age group more susceptible to disruptions in their routine and less susceptible to increased parental supervision during lockdown [89].

On the other hand, Tornese et al., in 2020, reported that adolescents who maintained regular physical activity during lockdown, in the form of at-home exercises for at least 3 h per week, showed a significant increase in TIR and thereby improved their glycemic control compared to adolescents who did not exercise during lockdown [90].

In contrast to the above-mentioned studies, an Italian study of 130 children, adolescent, and adult T1D patients using CGM reported no significant changes in glycemic control in adolescents during the period of lockdown as evidenced by comparable CGM metrics pre- and post-lockdown. As previously indicated, these findings have been attributed to teenagers’ growing desire for independence and autonomy, which often leads to a gradual reduction in family involvement during this stage of life [91].

It has been proposed in the literature that contributory factors to impaired glycemic control, such as unavailability of insulin and glucostrips, poor eating habits, and decreased physical activity, should be taken into account when planning for and managing chronic health conditions during pandemics in the future [79].

### Impact of telemedicine on T1D management

A large number of studies have attributed the use of telemedicine during lockdown to better glycemic control [80, 87, 92–94] and prevention of acute complications due to easier contact with the medical team [81]. Furthermore, parents reported that telemedicine made accessing clinical care more convenient during the pandemic, particularly for those living far from medical providers, and enabled both parents to attend appointments as many parents worked from home [80].

An observational multicenter study of 195 T1D patients < 25 years old reported a significant increase of 6.6% in TIR and a significant decrease of 3.4% in % time spent in hyperglycemia (180-250 mg/dL) following a telehealth visit during lockdown, either in the form of email, video, or telephone consultation [93]. Moreover, 86.7% of patients and their parents reported that they benefitted significantly from the visit, while 74.2% expressed strong interest in integrating telehealth in future care [93].

Predieri et al. reported a 3.5% increase in TIR, a 19% decrease in time below range (TBR, < 70 mg/dL), and a 5.56% decrease in TAR in a cohort of 62 pediatric T1D patients monitored via telemedicine during lockdown. Telemedicine reportedly contributed to improved glycemic control by enabling continuous monitoring and support to patients during lockdown and allowed healthcare professionals to offer guidance on maintaining a balanced diet, adjusting insulin dosages, and staying active at home [94].

In a follow-up study by Tornese et al., 2020, it was suggested that the continuous engagement with healthcare professionals and consistent oversight provided by telemedicine during lockdown may have played a key role in maintaining glycemic control both during and post-lockdown. This approach potentially enhanced patient adherence and monitoring of treatment regimens [92].

Similarly, an Italian real-life retrospective observational study of 139 children and adolescents with T1D reported a significant increase in number of contacts between parents and the medical team via telemedicine. This, in addition to increased parental supervision over insulin administration and diet management, likely contributed to improved metabolic control during the pandemic [87].

A large cohort study investigating the transition to telemedicine for diabetes care during the COVID-19 pandemic in 28,977 pediatric and adult populations with T1D reported that this transition did not affect access to care for most patients; however, non-English speaking pediatric patients seem to have shown significant reductions in telemedicine visits, suggesting a need for developing multilingual interfaces and ensuring integration of interpreters into telehealth visits [95].

Conversely, Tinti et al., 2021, reported no significant differences in glucose metrics, including TIR, TAR, TBR, and total daily insulin dose, between patients monitored via telemedicine and those who were not [84].

### Impact of diabetic technology on T1D management

Consistent with the findings of Conejero et al. [86], a study from Helsinki involving 245 children with T1D aged less than 16 years reported improved glycemic control during lockdown for those using CSII, compared to MDI, evidenced by improved TIR and lower average glucose levels. These improvements were particularly notable in adolescents and children using conventional sensor-equipped insulin pumps [96].

Contrasting results were however reported by others [94, 97]. For example, in a retrospective Italian study of 62 pediatric T1D patients, Predieri et al., 2020, found no significant difference in TIR between CSII and MDI users at the end of lockdown. Nonetheless, CSII users showed a reduction in time below range, which the authors attributed to greater parental involvement and the use of non-automated insulin delivery systems by the majority of patients, while MDI users experienced a decrease in time above range [94].

Literature regarding glycemic control in pediatric patients who used insulin pump therapy during the pandemic, compared to MDI, remains limited. Further studies should be conducted to determine the effectiveness of diabetes technology in maintaining or improving glycemic control in the context of the COVID-19 pandemic and lockdown.

## Conclusion

The COVID-19 pandemic affected multiple aspects of T1D in children and adolescents, including the incidence, presentation, and management of the condition. Most studies reported an increased incidence of T1D in the pediatric population during the pandemic and, while a causal relationship between SARS-CoV-2 infection and the development of T1D and/or the first clinical presentation of T1D has been hypothesized, further studies are needed to investigate this further. The delayed diagnosis and increased DKA frequency observed during the pandemic raises concerns regarding public knowledge of the early signs of T1D and highlights the importance of a timely diagnosis of this condition. Although numerous studies have associated periods of lockdown with improved glycemic control, reflecting better T1D management, others have shown that the pandemic negatively impacted patients living with this condition. The challenges that were faced during lockdown, such as reduced physical activity, worse dietary habits, shortage of insulin, and diabetes consumables, demonstrate the potential consequences of imposing restrictive measures on children and adolescents managing T1D. On the other hand, more parental involvement and the use of telemedicine appear to have had a beneficial effect on young patients managing this chronic condition.
